# The conceptualization of champions and their role in healthcare resilience

**DOI:** 10.1186/s12913-026-14858-8

**Published:** 2026-07-07

**Authors:** Eline Ree, Hilda Bø Lyng, Cecilie Haraldseid-Driftland, Veslemøy Guise, Birte Fagerdal, Heidi Dombestein, Hilde Valen Waehle, Siri Wiig

**Affiliations:** 1 SHARE – Centre for Resilience in Healthcare, Faculty of Health Sciences, University of Stavanger, Stavanger, N-4036 Norway; 2 Research and Development Department, Haukeland University Hospital, Bergen, Norway

**Keywords:** Champions, Resilience in healthcare, Healthcare quality, Facilitators, Capacities

## Abstract

**Background:**

Employees who demonstrate a deep commitment and passion for a specific cause or initiative within healthcare are often called champions, and they are increasingly recognized as key resources during implementation processes and quality improvement work. However, the inconsistent use of the champion concept in the existing literature addresses the need for more knowledge on how champions are conceptualized and identified in practice, and how they might contribute to resilience in healthcare.

**Methods:**

The study had a qualitative explorative design, in which 55 managers and healthcare staff from two Norwegian hospitals, five nursing homes, and one homecare service participated in an intervention aimed at strengthening resilience in healthcare. Data material from the implementation process was used, including focus group interviews before (*n* = 47) and after (*n* = 32) the intervention, and observation notes from the intervention workshops (15 workshops, 30 h observation). Thematic analysis was used.

**Results:**

Champions were conceptualized as individuals who see possibilities instead of obstacles, and who take on responsibility for finding and collecting required resources to solve challenges. They motivated others, and could possess both informal roles in facilitating social, relational and professional matters, and/or be assigned more formal roles as resource persons in specific fields or as facilitators for implementation processes due to their engagement. Scarcity of resources and lack of management support were important barriers in keeping and engaging champions. Leadership facilitation and support and to have a sound psychosocial work environment with a culture of openness and psychological safety were important enablers for champions.

**Conclusion:**

By being supported and acknowledged, champions may have the potential to contribute to resilient performance in healthcare. Champions are key resources for quality improvement work, implementation-, and change processes, and a sound organizational culture due to their great engagement and by going the extra mile for their organization. Managers have a key role in acknowledging and supporting champions and ensure that they are given opportunities to be a resource for the organization rather than having their efforts cover up system vulnerabilities such as scarcity of resources.

**Supplementary Information:**

The online version contains supplementary material available at https://doi.org/10.1186/s12913-026-14858-8.

## Introduction

In the dynamic, complex, and ever-evolving landscape of healthcare, employee enthusiasm and involvement play a key role in promoting positive change and for driving organizational success [1, 2]. Recognizing the importance of harnessing the power of employees, healthcare organizations have increasingly turned to facilitators and champions within their workforce to facilitate the implementation of innovative practices, improve patient outcomes, and enhance overall healthcare quality [2, 3]. In healthcare, champions are often defined as individuals who are especially dedicated to facilitate changes or innovations in their organizations [4, 5], or who have specific formal roles in leading or facilitating quality improvement initiatives [2]. Champions are furthermore described as individuals who demonstrate a deep commitment and passion for a specific cause or initiative within healthcare [6]. These committed individuals serve as role models, inspire, and motivate others, and go the extra mile to ensure positive outcomes in their areas of interest and expertise [3, 6, 7].

Champions are suggested to be an important capacity for resilient healthcare in their commitment to ensure positive outcomes [3]. Resilience can be defined in many ways depending on traditions such as psychology, engineering, and ecology [8]. We define resilience in healthcare as *“the capacity to adapt to challenges and changes at different system levels*,* to maintain high quality care”* [9 p6). We developed, chose, and use this definition as it positions resilience in the healthcare context and has a wider scope than the usual definitions within resilience engineering, on whose shoulders we stand and further build on [10]. The resilient healthcare perspective differs from the traditional ‘Safety-I’ approach to patient safety as its focus is on understanding and learning from what goes well in everyday clinical work, as opposed to the traditional focus on adverse events and outcomes [11, 12]. The Safety-I approach can be defined as *“the system quality that is necessary and sufficient to ensure that the number of events that can be harmful to workers*,* the public*,* or the environment is acceptably low”* [12 p. 1]. The Safety-I approach seeks to prevent errors and adverse events from occurring by learning from what goes wrong. Resilience is an essential part of Safety II, and the resilient healthcare perspective is often referred to as Safety II. This seeks to learn from what goes well, in addition to what goes wrong, and explores how the healthcare systems adapt when facing challenges. The system’s capacity to adapt is dependent on the multiple interconnected individuals within the system and how they act or react to ensure high quality healthcare services [13]. Healthcare professionals and managers in particular play a key role in enacting and supporting the adaptive capacity of the system [14].

Building on resilience research [12, 15, 16] and in support of the practical application of resilience in healthcare, research from a large Resilience in Healthcare (RiH) project have identified ten essential capacities for resilient performance across a wide spectrum of healthcare settings: structure, learning, alignment, coordination, leadership, risk awareness, engagement, competence, communication, and facilitators [3]. Facilitators serve as catalysts for change, helping organizations navigate the complexities associated with implementing new initiatives or improving existing processes [3]. Hence, the contributions of facilitators may be vital for creating a healthcare environment that adapts to evolving needs and consistently strives for quality improvement, which aligns to the definition of resilient performance on adapting to challenges and changes. According to Lyng et al. [3], champions and knowledge brokers are two specific types of facilitator roles that healthcare staff take on, which serve as an important capacity for resilient performance. Knowledge brokers facilitate the transfer of knowledge between colleagues and across boundaries as they are often exceptionally familiar with a given healthcare system and the stakeholders within it, while champions facilitate through their own actions, motivations, innovations, and initiatives [3]. Champions can be a resource for resilient performance which the organization may possess or may not possess and as such need to be identified, understood, supported and valued to act as a resource for resilient performance. While different concepts such as champions, knowledge brokers, facilitators, and opinion leaders have all been used interchangeably to describe the same or a similar concept [3, 5], the current study will use the term champions when referring to healthcare staff who might facilitate resilience in healthcare.

Previous research shows that champions might play an important role in several important outcomes in quality improvement projects, such as increased participation, job satisfaction among staff, and improved healthcare quality and patient outcomes [2]. Champions are found to be key for healthcare organizations to implement changes and adaptations in a range of different healthcare settings, such as the development of remote care or telehealth services [17, 18], midwifery practices [19], primary care [2, 20], and hospitals [21]. Based on previous research and the framework by Lyng et al. [3] which suggest that champions serve as a capacity for resilient healthcare, we may assume that champions might be an important means of promoting and supporting resilience in a healthcare system. Even though champions have been identified as a capacity for resilient performance [3], the role of champions needs further conceptualization to bring an understanding of how champions can contribute to promoting resilience in healthcare.

In this paper we explore the concept and role of champions in Norwegian healthcare services through the following research questions: (1) How are workplace champions conceptualized among healthcare professionals and leaders? and (2) What are the enablers and barriers to champions contributing to resilient performance?

## Materials and methods

### The resilience in healthcare (RiH) project

This article reports on a study that is part of the Resilience in Healthcare (RiH) project which is a 5-year (2018-24) research program that aims to enhance the quality of current healthcare systems by establishing a new and comprehensive Resilience in Healthcare framework [22]. As part of the goal to translate resilience in healthcare into practice, the researchers developed, implemented and tested a collaborative RiH learning tool in Norwegian healthcare services [23, 24]. The learning tool consisted of three key elements; (1) mapping the organizations’ capacity for resilient performance, (2) learning scenarios designed to encourage reflection and deepen the understanding of how resilience is enacted in everyday work, and (3) a resilience reflection list that employees could use in everyday work to foster ongoing reflection. The learning tool was implemented through three workshops that were structured around these elements, ensuring alignment with the learning tool’s functions. In between each workshop the participants were given a specific task where they needed to engage a group of healthcare staff at their unit in collaborative resilience reflections using the different elements of the learning tool. The evaluation of the intervention is ongoing, and although some results are available [23], the main part of the implementation process and results will be published in upcoming papers. The current paper uses data from workshops and focus group interviews conducted during the implementation process but is not part of the evaluation of the intervention.

### Design and setting

This study has a qualitative explorative design, using data from the implementation and evaluation phase of the resilience learning tool intervention, which included semi-structured focus group interviews pre- and post-intervention and observation data gathered during the three workshops facilitated by researchers. This approach allowed us to gather valuable insights from managers and healthcare personnel on how champions contribute to organizational resilience.

The study was conducted in six Norwegian nursing homes, two hospitals, and one homecare service. The sites were in five different municipalities of varying sizes and included urban and rural locations (Table 1). Five of the nursing homes are part of a private non-profit enterprise whose purpose is to develop and operate nursing homes on behalf of the public. Any operating surplus is utilized for activities within the company’s purpose, and the enterprise does not aim to generate financial profits for its owner. These five nursing homes are in a large urban municipality, while the last nursing home is located in a smaller, rural municipality and is publicly owned and operated. The homecare service and the two hospitals are also public.

**Table 1:** Setting and data collection

Municipality location & number of inhabitants	No.	Setting	Units represented	No. of participants	Pre-intervention FG^a^	WS1^b^	WS2	WS3	Post-intervention FG
Rural area approx. 10.000	1.	Homecare services	Homecare services	2	2	2	2	2	2
Rural area approx. 25.000	2.	Nursing home	Short-term care, rehabilitation, Long-term care	4	2	3	2	2	2
Urban area > 600k	3.	Nursing home	Cross sectional teams (Safety and quality unit, occupational therapist, activity coordinator), Long-term care, Resident secure units	9	9	7	2	1	1
	4.	Nursing home	Long-term care Resident secure units	11	8	10	10	8	8
	5.	Nursing home	Long-term care Day care services	7	5	3	3	2	2
	6.	Nursing home	Resident secure units	2	2	0	0	0	0
	7.	Nursing home	Long-term care	2	2	0	0	0	0
Urban area > 250k	8.	Hospital	Neurology	11	2	2	2	2	2
			Cancer care 1		2	3	2	3	3
			Cancer care 2		3	3	2	3	3
			Cancer outpatient clinic		3	3	3	3	3
Urban area > 130k	9.	Hospital	Therapy department (physiotherapists, occupational therapists, social workers, and speech therapists)	7	2	2	2	2	2
			Mental health		1	0	0	0	0
			Nutrition unit		1	1	1	1	1
			Quality department		3	3	3	3	3
Total:				**55**	**47**	**39**	**31**	**32**	**32**

^a^ FG= Focus groups, ^b^ WS= Workshops

The healthcare system in Norway is semi-decentralized where four Regional Health Authorities are responsible for specialist healthcare (i.e., hospitals, outpatient clinics), and the municipalities are responsible for the primary healthcare and social services (i.e., nursing homes, homecare services, General Practitioners) [25]. About 85% of the healthcare services are publicly funded through central and local governments, as well as the National Insurance Scheme, representing the largest proportion in Europe. Private health financing primarily relies on out-of-pocket payments made by households, with a significant portion allocated to pharmaceuticals, dental care, and long-term care. Although the number of Norwegians with private health insurance has risen in the last decade, its impact on health financing remains minimal [25].

### Recruitment and participants

The sites were selected using a combination of purposive and snowball sampling. Initially, both sites and participants were recruited through the researchers’ professional network, followed by obtaining formal approval from site management. A key consideration in the purposive sampling strategy was to include diverse healthcare settings across both primary and secondary care. This approach ensured that the tool’s applicability across different contexts could be explored. In total, 55 individual participants took part in the intervention. The number of participants involved in each data collection activity—pre- and post- focus group interviews and workshops — varied according to staff availability at the time of each session. Most participants were managers at different levels (i.e., department/ward managers, quality leaders, culture manager, group leaders, assistant department leaders, chief of staff). In addition, healthcare staff without leadership responsibilities were also represented, such as registered nurses, healthcare workers, assistants, an occupational therapist, a physiotherapist, and professional development nurses. Different wards and departments were represented both in the hospitals, nursing homes and homecare services (Table 1). We consider the sample of healthcare professionals and managers from various healthcare settings highly relevant for exploring the aim of this study, as they can provide different experiences, nuances and perspectives on how champions contribute to resilience in Norwegian healthcare services.

### Data collection

Eight semi-structured focus group interviews (*n* = 47) were conducted before the intervention and six semi-structured focus group interviews (*n* = 32) were conducted after the intervention. Most of the participants in the post focus group interviews were the same as those who participated before the intervention. Focus group interviews [26] were chosen as the participants were colleagues and knew each other and could discuss experiences concerning work practices. The researchers in the project had different backgrounds (i.e., safety science, nursing, psychology, quality advisor) and were all experienced in qualitative methods and implementation.

Two of the pre-intervention interviews were conducted at the participants’ workplaces, while the rest were conducted digitally. All post-intervention interviews were conducted at the participants’ workplace following the third and final workshop. The interview guide before the intervention consisted of themes related to their daily practice considering the ten capacities for resilient performance: structure, learning, alignment, coordination, leadership, risk awareness, engagement, competence, facilitators, and communication [3]. In the post-intervention focus groups, the questions were about participants’ experiences with the learning tool and the implementation process. All focus group interviews lasted 60–90 min and were audiotaped and thereafter transcribed verbatim by a research assistant or professional transcription services. The researchers who conducted the interviews verified the accuracy of the transcriptions. Two researchers were present in all focus group interviews, acting either as moderator or secretary. Additionally, 15 workshops were held throughout the course of the intervention period, during which a total of 30 h of observations were conducted. Participants from all sites attended three workshops, with some nursing homes participating jointly due to their shared location. Two to four researchers facilitated each workshop, and their observation notes are part of the data set. The observation notes included notes about who participated in each respective workshop, group dynamics, how the participants talked about and understood the learning tool and the concept of resilience in healthcare, how they had used the tool in their workplace, and how they reflected on and collaborated in groups during the implementation process.

### Analysis

Thematic content analysis was used to analyse the data, as the method is suitable for identifying patterns of meaning across the data material [27, 28]. Themes were derived inductively following Braun and Clarke’s [27, 28] six-step approach to thematic analysis: (1) Transcripts and observational data were thoroughly read to become familiar with the data and establish a general understanding of the content; (2) quotes in the text of relevance to the research questions were then identified, labelled with codes, and organized into an analysis matrix; (3) once all data were coded, the codes were sorted into preliminary themes. The sorting of codes was done collaboratively with all the co-authors; (4) the themes were subsequently cross-checked against the coded meaning units and the entire dataset, which provided a deeper understanding of the overall picture and allowed for adjustments to the themes as well as dividing them into sub-themes; (5) the final labelling of each theme was determined and verified to ensure they addressed the research questions and reflected the experiences in the original transcriptions; and (6) the results were described with quotations, as is detailed in the article. See Table 2 for an excerpt of analysis matrix with quotes, codes, and preliminary themes.

**Table 2:** Excerpt of analysis matrix with quotes, codes, and preliminary themes

Quotes	Codes	Preliminary themes
Then (to take care of the champions) you need to have a good dose of empathy, humanity and competence around that, I think. And (…) “seeing” the individual, and then giving the individual responsibility for what they have, for what they are “passionate” about	Certain characteristics are necessary to take care of the champions	Enablers for champions
And, you must know the staff, so to say. It is important to be familiar with them, and to know each one of them.	The importance of managers knowing their staff	
Yes, one gets, in a way, that openness to develop. But, also, being “looked after”. It’s… having both of them…	Getting possibilities to develop but also being looked after	
So, they are not recognized. And their competence is not recognized.	Managers do not recognize champions’ competence	Barriers for champions
I believe that we have many champions. And, then the challenge is managing and keeping them there. Because, it’s busy. And time…. you have to find time, if you want to do something, immerse yourself in something.	Challenging keeping champions because of resource scarcity	

## Results

The analysis resulted in four main themes with associated sub-themes. These results are elaborated below.

### The conceptualization and identification of champions in everyday work

#### Conceptualization of champions

Champions were described by the participants as persons that see opportunities instead of obstacles, and who take on a responsibility for finding and collecting the resources needed to solve challenges. They were also described as “yes-persons” that helped and guided others, and that had great importance for the organization. Several participants described champions as informal leaders that were particularly good at their job and who people listened to. Individuals described as champions were often the ones to welcome and take care of new staff members, and those people went to for help and advice in the face of obstacles or challenges. These individuals were the ones who went the extra mile in everyday work, both for the patients, for colleagues and for the organization, and they often made it seem effortless. An example of these individuals’ dedication was that they could participate in digital morning meetings during their days off or send emails or take courses outside of working hours without getting paid. Furthermore, the champions could for example facilitate a pleasant time for patients in the nursing home in only two minutes, something which for others would take much more effort, described as follows by a nurse:

*I think what characterizes such a person is someone who sees opportunities*,* and not limitations. (…) Someone who takes on responsibility *(Nurse, nursing home 7).

There was a debate in two of the focus group interviews concerning the use of the term champion about staff in healthcare settings, and other concepts were suggested such as engagement, engaged employees or enthusiasts. Some associated the concept of champions more with voluntary work, and several argued that everyone working in healthcare was a champion in different ways and that all employees contributed somehow. Some of them, they said, were louder and more visible, while others worked more quietly and did a lot of “hidden work” contributing to high quality services. It was perceived to be an important management task to also notice the “quieter” champions. Some were concerned that giving attention to the few obvious champions would be at the expense of others who were as important for the organization and contributed in other, less visible ways.

#### Identification of champions

Most participants agreed that champions were easy to notice at work due to their motivation, engagement, and enthusiasm for specific fields. The champions were also identified through their efforts in engaging and motivating those around them, as illustrated by this quote:

*The champions usually identify themselves. They stand out. In a good way* (Nurse, homecare services 1).

Although most champions were identified through their actions or through colleagues noticing and talking about them, some managers also took the effort to map their staffs’ fields of interest either through personnel meetings or employee conversations. When employees were given responsibility for specific fields of interest, their engagement and motivation increased, and some became formal workplace champions within those fields. Others talked about the importance as a manager to be present “on the floor” to be able to see and identify champions among staff members. For example, an administrative department nurse worked 20% of her position as a clinical nurse in one of the nursing home wards in her organization. This allowed her to notice that some of the employees did extra little things in addition to their regular tasks to make the days better for the patients or to respond to or adapt to things happening in the course of everyday work. Managers could also identify champions through colleagues talking positively about them, as one manager described it:

*They show themselves through different channels*,* such as someone coming to me saying: You know what*,* he/she is absolutely amazing! He/she has so many ideas!* (Assistant department nurse, hospital 8)

### The role and importance of champions in healthcare services

#### Champions being assigned formal roles as resource persons or coordinators

Most of the participants emphasized the important role of champions in everyday work of their healthcare organization, both for patients, the psychosocial work environment for staff, and for ensuring the quality of services in the organization. Champions often served as resource persons or coordinators, both in nursing homes, homecare services, and in hospitals. As resource persons they had responsibility for specific fields such as wound care, cancer care, welfare services, or nutrition, and could sometimes possess coordinator roles such as dementia coordinators, cancer coordinators or welfare coordinators. For example, champions in one of the nursing home departments had pulled some strings to set aside resources for a coordinator position. According to the participants, this position was necessary to ensure good quality work within the department. The person in the coordinator position was a champion who had an overview of the department, ensured good information flow, served as a link between the department, homecare services, and the specialist healthcare services, as well as being an overall resource person that other employees went to with challenges and questions. The managers agreed that assigning formal roles to champions, in a field they were already engaged in, both increased motivation among the champions and made the system and services better. As one manager pointed out:

*I would say that we always have resource persons*,* and then we have some champions. And it is amazing when these two roles can be combined* (Assistant department nurse, hospital 8).

Whenever staff had questions related to specific professional fields, managers could refer them to the designated resource persons or coordinators for that area. Many managers also mentioned having readily available lists of these individuals for staff to consult. The resource persons had priority to attend courses relevant to their resource area but were then responsible for informing and teaching colleagues what they learned on the courses, for example by giving presentations and lectures during lunch breaks or staff meetings. In this way the champions were important as knowledge brokers in the work force.

#### The roles of champions in facilitating social, relational and professional matters

Many spoke about having several champions at their workplace who each had different fields in which they had a special engagement and commitment, without necessarily being assigned formal roles as resource persons or coordinators. This could be in different professional fields (e.g., quality and patient safety, cancer care, pain relief, nutrition), or social engagement both for patients and colleagues (e.g., planning social events), and relational engagement.

Several examples showed that staff in different ways went the extra mile for patients and residents, such as a qualified make-up artist who gave manicures to and put make-up on the residents. Another played piano for them, while some others took the residents out on walks and spent time with them, drinking coffee and having good conversations. A nursing home manager emphasized that it was important to not only acknowledge the professional healthcare workers with high competence in clinical matters and exemplified this with an unskilled employee that had excellent cooking skills and served as a champion by spending a lot of time preparing good meals for the residents and involving residents that were fond of baking in the kitchen. These were all things that the residents really appreciated.

Others were social champions at the collegial level and made an extra effort regarding the psychosocial work environment, such as celebrating birthdays and arranging social events. The champions’ engagement often spread to other colleagues and thus had a positive effect on the work environment.

Several participants talked about professional champions which were staff with high engagement in clinical matters. For example, during the COVID-19 pandemic, an employee at a hospital intensive care unit took special responsibility for collecting and spreading information among staff. She spent a lot of time reading up on all the new information and guidelines about the pandemic and made procedures based on this. Other champions were often engaged in research projects and other improvement projects or interventions in the organization.

Champions were often people whom the staff listened to and respected, and therefore had a lot of informal power to affect and change the organizational culture. Hence, they were important when implementing changes or projects to engage staff through these processes, which some participants believed increased the chances of successful implementation. Furthermore, they were considered important drivers in these processes due to their capacity to adapt to changes and find solutions in the face of challenges. A manager expressed this as follows regarding one of her employees during an implementation process:

*We had some ideas about how to do it*,* and it didn’t turn out that way*,* but what’s wonderful is that we have people who can say ‘I’m just doing it anyway. Because that’s how it is to work in homecare*,* so then it is absolutely amazing that [name of employee] just pulls it off anyway *(Department manager, homecare service 1).

### Barriers to keeping and engaging champions

#### Scarcity of resources

The most common barrier to taking care of and ensuring the engagement of champions was the scarcity of resources. Although champions were perceived as important resources in the workforce, they often had ideas and initiatives that were challenging for the managers to accommodate due to a lack of resources such as time and money. Some also experienced that the champions were impatient and lost their enthusiasm when things took time to come to fruition, as is often the case in the healthcare services. Furthermore, a large workforce often made it difficult for managers to get to know each of their staff members individually, especially if there were many part time workers. The large workforce also made it more challenging for them to identify and keep champions in their workforce.

*There is very little “room for action” here*,* as most of the time is taken up by daily operations. Referrals keep coming*,* and you must take care of them*,* right? (…) So*,* there is little room to do something different or extraordinary. Many employees want to do a lot. But then… it doesn’t work*,* because there is too little time *(Quality and safety advisor, hospital 9)

Some of the managers said it was a challenge in cases where the champions were interested in areas that did not benefit the organization. The majority however appreciated the champions as important resources at their workplace, but still found it challenging to take care of them in the busy everyday work setting. This was especially the case in workplaces with several champions who often came up with new initiatives and ideas independently of each other, so that the managers had to prioritize between their differing suggestions.

*I believe that we have many champions. And*,* then the challenge is managing and keeping them there. Because it’s busy. And time…. you must find time*,* if you want to do something*,* immerse yourself in something* (Ward manager, hospital 8)

They saw it as a management task to keep the champions’ sparkle and enthusiasm and said that if champions too often experienced not being heard and accommodated regarding their initiatives, they would eventually burn out. Some participants pointed out the challenge of having only one or two champions acting as resource persons or leaders of projects, as implementation then was dependent on these persons, and therefore vulnerable if they were off work for different reasons.

#### Lack of management support

Another barrier was not being listened to or appreciated by the managers. Some described champions who contributed far outside of their regular work tasks without being recognized by their managers. A few managers expressed annoyance with some of their staff who always came up with new ideas and new ways of organizing the services, as they did not have time to deal with their suggestions and perceived them as nagging. In one nursing home, staff said that the managers did not see or acknowledge champions who went the extra mile at work or were particularly passionate for certain areas. For example, two champions had been assigned by management to take part in a research project. However, the managers did not support them during the project, there were changes in the management group, and the management group changed its focus on what they chose to prioritize. This left the champions alone without sufficient time and resources to carry out the project as planned. Another example of poor management came from an employee who expressed that they had been met with the statement *“if you don’t like it here*,* then quit”* by a manager (nursing home 2) when suggesting changes at their workplace.

### Enablers for champions to function as a capacity at work

#### Leadership facilitation and support

Leadership was the most important enabler for champions to function as a capacity in the healthcare organization. Managers had several responsibilities, including identifying the champions, keeping up their engagement and passion, and preventing burnout. Leadership involvement was also important, such as being present in the department and engaged in ongoing projects, noticing the staff’s efforts for patients and the organization, and involving employees in matters that are of importance to them. Managers facilitated for and supported champions in several ways, including assigning them formal roles as resource persons or coordinators in their field of interest, and to be open and enthusiastic to their suggestions and take them seriously. Knowing the staff well was an important prerequisite to perform these tasks well. One important aspect of a good quality healthcare service was having the right mix of people according to competence and interests. Knowing their staff made it easier for managers to suggest people for specific tasks based on their interests and competence, provide extra challenges for those they knew appreciated it, and put together well functioning teams based on their competence and interests, as illustrated in this quote:

*And then there is this thing about putting together good teams. As a manager*,* you must get to know your employees because then you have the opportunity to succeed*,* and we as managers have to take on that job. Because I know from time to time that I am sitting here with the most wonderful team*,* we can move mountains. (…) Then you know who the resource person is*,* who is the one who really pulls the load. They see that we can actually do this*,* and then there is something about having role models. So*,* this is competence*,* resource persons*,* those who are driving forces…. And I have to be very interested in them* (Quality manager, nursing home 4).

It was considered important to give champions opportunities to develop and to adapt their work tasks as much as possible to make room for the champions’ initiatives. When champions engaged in implementation processes or projects it was seen as significant to have support from management throughout the process, to keep their enthusiasm up and to ensure successful implementation. It was not enough that the managers’ expressions were positive, they also had to help set aside dedicated time for the champions to actually be able to carry out their projects. For example, one of the managers said that if an employee made an extra effort but was scarce on time, they could facilitate for them by stepping in and helping the employee with some of their regular work tasks, making room for the champion to finish the special tasks they were engaged in.

The support of champions required that managers possessed empathy, competence, and the ability to see individuals and give them responsibility for the things they were passionate about. One of the healthcare workers exemplified this by talking about her own manager, who had the ability to boost people, but also to regulate working pace when necessary. The latter was important to reduce the risk of burnout among champions.

Managers had a responsibility for providing their employees with what they needed to thrive in their work, which differed based on personality and interests, as well as what they needed to gain and retain their motivation. Individual adaptation was therefore important, as illustrated by this quote by a manager:

*Champions are a real mixed bag of people. So*,* the important thing is to manage and bring out the best in each person according to what they are passionate about* (Nurse, nursing home 2).

#### A sound psychosocial work environment, psychological safety, and openness

Having a sound psychosocial work environment with a culture of openness and psychological safety was an important enabler for champions, and managers were key in creating these conditions. In one nursing home, managers had implemented an initiative called ‘Valuable’ where the staff nominated each other approximately once a quarter for making an extra effort, to create engagement and motivation among staff. The managers were not allowed to vote, and the colleague with the most votes received a diploma. Talking positively about each other, cheering for each other, and noticing colleagues’ resources, competence and skills were considered encouraging and made it easier for champions to thrive and maintain their enthusiasm.

Several managers said they worked to create a safe work environment, by being present and inclusive, open to and respectful of new ideas, and having a good dialogue with staff. Many practiced an open-door policy, joined staff lunches, and worked to create a sense of community among staff. Not all ideas, initiatives and wishes were possible to accommodate, but it was important that leaders then explained the reasons why and maybe tried to find alternative solutions together with staff. Being open to new suggestions and ways of doing things and listening to each other was positive for staff motivation and engagement and had a general positive effect on the work environment.

*In the department*,* I feel that there is openness to bring forth new ideas and solutions. We are solution-oriented*,* and we listen to each other. I know that the Managing Director is very receptive to new ideas*,* so it’s possible to send an email* (Ward manager, hospital 8).

## Discussion

This study explored how champions in Norwegian healthcare services were conceptualized and identified in everyday work, the different roles they have in the organization, barriers to keeping and engaging champions, and enablers for champions to function as a capacity for resilient healthcare as perceived by healthcare staff and managers.

### Conceptualizations of champions

Champions were described as persons who see possibilities instead of obstacles; who take on a responsibility for finding and collecting the resources needed to solve challenges; who help and guide others; and who go the extra mile at work. These qualities align with what can be defined as the soft dimensions of care quality, which staff in nursing homes and homecare services have identified as essential for delivering high quality healthcare services [29]. Similar findings have also been reported in hospitals across Europe [30]. Aase et al. [29] found that healthcare professionals in nursing homes and homecare services emphasized soft dimensions as essential when conceptualizing high-quality care. These included a sense of professional pride in having enough time to be available for patients, providing personalized care, engaging in ethical reflections with colleagues, and treating patients with dignity—even when this meant going beyond their formal job descriptions, such as ensuring patients’ rooms were welcoming.

In relation to our study, these soft dimensions align with the idea of going the extra mile and prioritizing patient-centered care, and may also explain why some participants in our study advocated for seeing most healthcare personnel as some kind of champion; it is part of their professional pride to always treat their patients with dignity and be willing to go the extra mile. These soft dimensions should receive greater attention in both research and quality improvement work in healthcare, as we see major challenges related to workers facing burnout due to not feeling able to provide healthcare services according to their professional standards, and to healthcare services’ understanding of how this can be done [31, 32].

### Champions’ contributions to resilience in healthcare

**Fig. 1:**
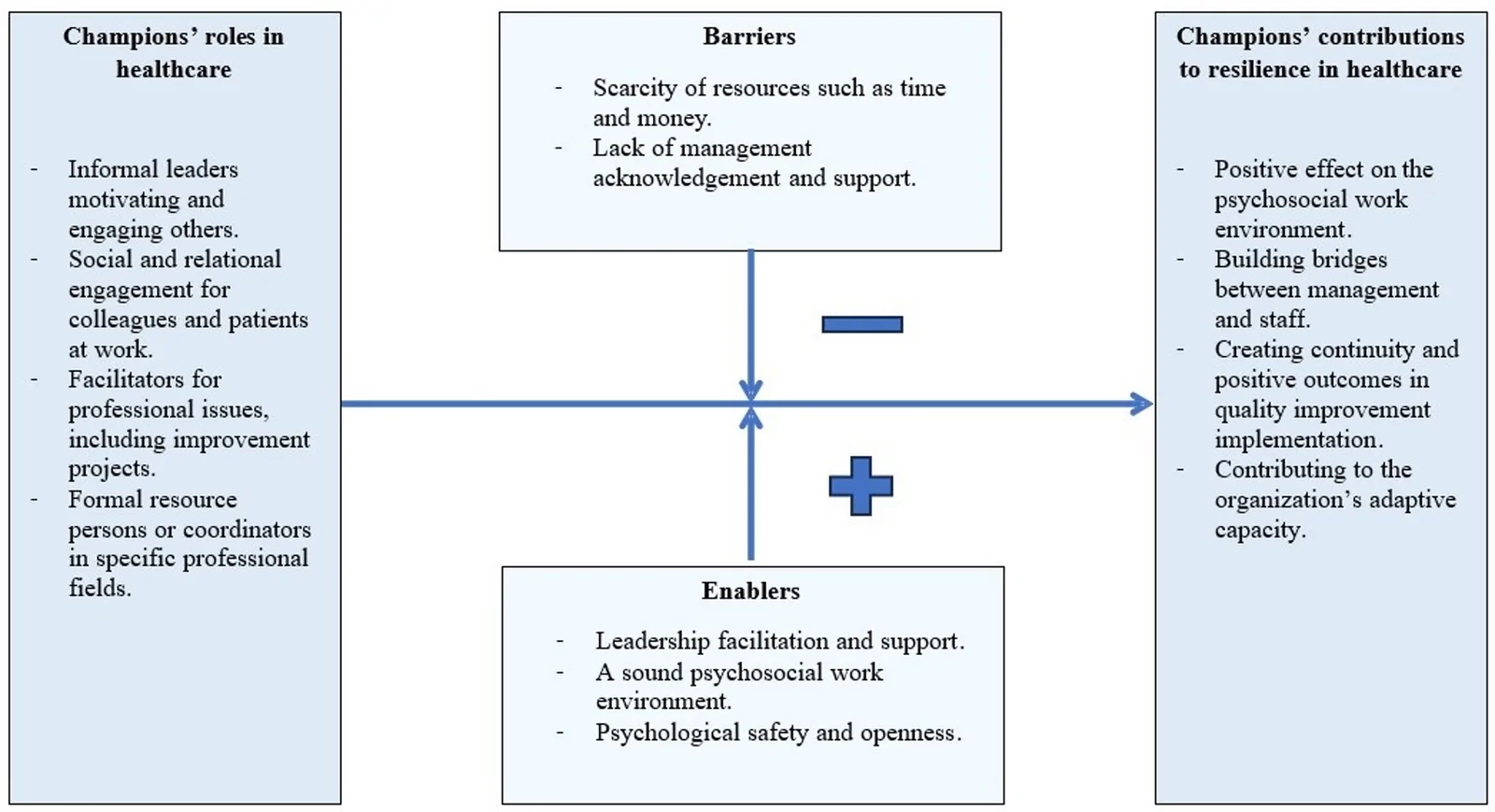
The role of champions for resilience in healthcare

In line with the conceptual framework suggested by Lyng et al. [3], our study indicates that champions might function as a capacity for resilience in their organizations, by facilitating quality improvement projects, contributing to necessary adaptations and changes, and making the extra effort in everyday work, both for patients and families, for colleagues and for the organization. By possessing informal power and being individuals that colleagues listen to, champions have the potential to serve as important actors in changing organizational culture and implementing changes to improve quality, and they can bridge the gap between staff and management in change processes or implementations (See Fig. 1). An important finding was the expressed benefits of using champions in more formal roles such as resource persons or coordinators, as their engagement could then be exploited in a positive way for the organization. The champions were also perceived as important facilitators in quality improvement projects. This is in line with several previous studies where champions are often used as part of an implementation strategy [2, 5], or as important drivers for change processes and innovations in healthcare [5, 17, 18]. A recent systematic review does however conclude that there is not sufficient evidence to support the positive role of champions on innovation use and outcomes (5). There has been a marked increase in research related to champions, but most of the research is about the champion’s role for implementation success (4). Our study adds to this by showing how champions might serve as an important resource for resilience in healthcare organization not just in their formal roles, but also through their informal leadership roles in the context of everyday work. Hence, while champions share similarities with early adopters in implementation science, their role extends beyond quality improvement initiatives to actively fostering resilience by mobilizing resources, strengthening networks, and supporting organizations in navigating challenges. Champions in our study were assigned their roles as resource persons and coordinators based on their fields of interest, which is in line with the recommendations by a previous study on the formal and informal roles of champions [33]. The study by Luz et al. [33] argues that healthcare organizations would benefit from allowing champions to choose which projects and innovations they would like to facilitate based on their interests and engagement.

### The importance of supporting champions

Our study suggests several ways in which to encourage informal champions to emerge and make them thrive, including leadership support and involvement, and a sound psychosocial work environment with a culture of psychological safety and openness to expressing new ideas. The important role of leaders in supporting, engaging, and facilitating for their employees and teams are echoed in previous studies [2, 34, 35]. Our findings align with those of a recent study by Fagerdal et al. [34] who demonstrates managers’ role in enhancing the adaptive capacity of their hospital teams by ensuring that the teams get the competence they need, by balancing workload assignment with the needs of their staff, and by practicing relational leadership with high levels of support. The study findings furthermore align with a suggested definition of managerial resilience as *“the strategies managers use to engage people in collaborative and coordinated processes that adapt*,* enhance or reorganize system functioning*,* promoting possibilities of learning*,* growth*,* development and recovery of the healthcare system to maintain high quality care”* [36 p. 276]. As such, managers’ support of champions can and should be considered part of their strategies to promote resilience in healthcare. Strategies for promoting resilience are in line with general leadership approaches used to improve healthcare quality [37, 38], and enhance psychological safety [39]. We encourage further research into this field as managers’ role in resilience has been pointed out as a crucial element in enabling resilient performance in healthcare [14, 36, 40]. However, how this role is enacted in diverse settings, which tools leaders use (or lack) in these processes, and how they do individual adaptations to support workers, are all elements in need of being investigated to learn and improve skills and competences among future leaders in healthcare.

### The flipside of the coin regarding champions

This study shows how champions are perceived as an important resource in healthcare organizations by healthcare staff and managers, with a high potential to help promote organizational resilience. Champions seem to have a high capacity to adapt and are drivers for change processes in the organization they work in. However, not all adaptations are positive [41–43], and the short-term adaptations and the extra effort made by champions also has the potential for making the organization vulnerable. Hendy and Barlow [18] questioned the role of champions in organizational change processes as they could have a negative impact on progress in later stages of implementation if they did not identify with the new circumstances. Another study found that the uptake and sustainability of telehealth services was vulnerable to cessation if champions as key drivers of the process lost interest or moved on [17]. This vulnerability was evident in our study, where participants described implementation processes which became seriously hampered if the champion went on sick leave or left the organization. By going the extra mile and finding ways to adapt when the system does not work as it should or in case of resource scarcity, champions might cover up system vulnerabilities which in a worst case can become a barrier for resilience in healthcare [42, 44]. In addition, it might lead to negative consequences for the champions enacting resilience, such as stress and burnout [7]. This shows the importance of managers noticing and being aware of the work champions do, and to use them as an extra resource rather than as a means to cover up vulnerabilities in the organization.

## Strengths and limitations

The main strength of this study is the large and diverse sample, providing different perspectives and nuances in the data material. Due to the qualitative nature of the study the results cannot be generalized, but the large and diverse sample including both nursing homes, hospitals and homecare service enhances the transferability of the results to other similar healthcare settings. Despite the small sample from home care services, the experiences shared by home care participants were largely consistent with those from other healthcare settings, supporting the relevance of the results. A limitation is that the focus group interview guides were designed for use in the overall project and the implementation process and not for this specific study investigating the concept of champions, meaning that only parts of the vast data set was used for this study. However, there was sufficient rich data included to conduct a responsible analysis and answer the research questions in this study. We had a specific research question, our sample had relevant experiences to share, the dynamic in the focus group interviews was good with fruitful discussions, and the study was guided by relevant theory, all of which contributed to sufficient information power to respond to the study research questions [45]. To reduce the risk of social desirability bias, we emphasized the importance to the participants of sharing both positive and negative experiences, and that all experiences and perspectives were welcomed. Furthermore, reflexivity and bracketing were important aspects of our approach to minimize bias. As a diverse research team with different backgrounds and experiences, we held regular meetings throughout the project in which we actively challenged each other’s preconceptions and engaged in reflexivity. This allowed us to reflect on how our own perspectives might influence data collection and interpretation. Additionally, we employed bracketing by consciously setting aside our assumptions before and during interviews and data analysis to ensure an open exploration of participants’ perspectives.

## Conclusion and implications

This paper explored how healthcare professionals and managers in Norwegian nursing homes, hospitals, and homecare service conceptualize champions and their role for resilience in healthcare services. The study shows that champions may have a key role in supporting resilience through being crucial resource persons during quality improvement processes, adapting to changes, and going the extra mile for the organization they work in. The study also highlights the importance of managers as enablers for champions to serve as a capacity for resilient healthcare, by identifying, supporting and encouraging champions, and facilitating a sound psychosocial work environment with a culture of openness and psychological safety.

These findings have practical implications for healthcare leaders, policymakers, and practitioners by showing how champions can be leveraged as a strategic asset in the pursuit of a more resilient, innovative, and responsive healthcare system. It is prudent to acknowledge and support champions and allow organizations and their leaders to take advantage of these resources in practice. This does not mean creating an unfair working environment, favoring some employees over others. Rather, it means seeing people and promoting humanity and motivation in the work environment and adapting working conditions to support everyone, both champions and their colleagues, as they might need diverse approaches to thrive and to support system resilience in everyday work.

## Supplementary Information

Below is the link to the electronic supplementary material.

Supplementary Material 1

Supplementary Material 2

## Data Availability

Data material can be made available upon reasonably request to the corresponding author of this manuscript.

## Abbreviations

RiH: Resilience in Healthcare
